# VDAC–Tubulin, an Anti-Warburg Pro-Oxidant Switch

**DOI:** 10.3389/fonc.2017.00004

**Published:** 2017-01-23

**Authors:** Eduardo N. Maldonado

**Affiliations:** ^1^Department of Pharmaceutical and Biomedical Sciences, Medical University of South Carolina, Charleston, SC, USA; ^2^Hollings Cancer Center, Medical University of South Carolina, Charleston, SC, USA; ^3^Center for Cell Death, Injury and Regeneration, Medical University of South Carolina, Charleston, SC, USA

**Keywords:** cancer metabolism, erastin, glycolysis, mitochondria, oxidative stress, tubulin, voltage-dependent anion channel, Warburg effect

## Abstract

Aerobic enhanced glycolysis characterizes the Warburg phenotype. In cancer cells, suppression of mitochondrial metabolism contributes to maintain a low ATP/ADP ratio that favors glycolysis. We propose that the voltage-dependent anion channel (VDAC) located in the mitochondrial outer membrane is a metabolic link between glycolysis and oxidative phosphorylation in the Warburg phenotype. Most metabolites including respiratory substrates, ADP, and Pi enter mitochondria only through VDAC. Oxidation of respiratory substrates in the Krebs cycle generates NADH that enters the electron transport chain (ETC) to generate a proton motive force utilized to generate ATP and to maintain mitochondrial membrane potential (ΔΨ). The ETC is also the major source of mitochondrial reactive oxygen species (ROS) formation. Dimeric α-β tubulin decreases conductance of VDAC inserted in lipid bilayers, and high free tubulin in cancer cells by closing VDAC, limits the ingress of respiratory substrates and ATP decreasing mitochondrial ΔΨ. VDAC opening regulated by free tubulin operates as a “master key” that “seal–unseal” mitochondria to modulate mitochondrial metabolism, ROS formation, and the intracellular flow of energy. Erastin, a small molecule that binds to VDAC and kills cancer cells, and erastin-like compounds antagonize the inhibitory effect of tubulin on VDAC. Blockage of the VDAC–tubulin switch increases mitochondrial metabolism leading to decreased glycolysis and oxidative stress that promotes mitochondrial dysfunction, bioenergetic failure, and cell death. In summary, VDAC opening-dependent cell death follows a “metabolic double-hit model” characterized by oxidative stress and reversion of the pro-proliferative Warburg phenotype.

## Introduction

### Warburg Metabolism: A Phenotype of Proliferating Cells

The Warburg phenomenon, named in honor of Otto Warburg’s work on lactic acid production in tumors, is a metabolic phenotype characterized by enhanced glycolysis and suppression of mitochondrial metabolism even in the presence of physiological levels of oxygen (1, 2). Warburg also postulated that respiration in the grana (mitochondria) of cancer cells was irreversible but not completely damaged and that permanent defective respiration originates cancer. According to Warburg, cells with damaged respiration compensate the lower energy production in the grana with increased aerobic fermentation (conversion of glucose to lactic acid). Only those dividing cells that increase fermentation enough to compensate for defective respiration would become cancerous (2). Impaired respiration as a driver of the glycolytic phenotype was immediately challenged by data from Weinhouse and others demonstrating both high glycolysis and oxidative metabolism in cancer tissues (3). Since the initial work of Warburg, enhanced glycolysis has been shown in nearly all tumors and cancer cell lines studied. Further investigations showed that mitochondria in cancer cells are functional as determined by measurements of mitochondrial membrane potential (ΔΨ), ATP, and NADH production among other parameters (4–10).

Although overall ATP production in tumors is contributed both by glycolysis and oxidative phosphorylation (OXPHOS), mitochondrial contribution to total ATP is always lower in cancer cells compared to differentiated cells. Differentiated cells produce about 95% of the total ATP by OXPHOS and the remaining 5% through aerobic glycolysis. In cancer and other proliferating cells, glycolysis accounts for 20–90% of total ATP production with the remainder contributed by mitochondrial oxidation of pyruvate, fatty acids, and glutamine (6, 11). A highly glycolytic phenotype has been associated with a high rate of cell proliferation (11–14). The “glucose avidity” of tumors is the foundation for the positron emission tomography of the glucose analog ^18^fluorodeoxyglucose to diagnose primary tumors, recurrences, and metastases (15). Noticeably, the bioenergetics of tumor cells is different among tumor types but even in cells from the same type of tumor. Subsets of cells with either high glycolysis or high levels of OXPHOS have been identified in gliomas (16, 17) and large B cell lymphomas (18) opening a new perspective to understand the consequences of different bioenergetic profiles in cancer metabolism.

A possible physiologic and evolutionary advantage of the Warburg phenomenon could be the provision of enough energy for frequent cell division. However, glycolysis generates only 2 moles of ATP per mole of glucose, whereas full oxidation of 1 mole of glucose to CO_2_ and H_2_O in mitochondria generates 29–32 moles of ATP, as estimated by different methods (19). The low efficiency of ATP generation through glycolysis has been considered to be offset by the increase in the rate of glycolysis making the overall production of ATP in proliferating cells higher than in those that do not proliferate (20). Interestingly, the amount of ATP necessary for biosynthesis is lower than the energy requirements for basal cellular processes that maintain cell homeostasis making unlikely that ATP be rate limiting for cell proliferation (21).

**Figure 1:**
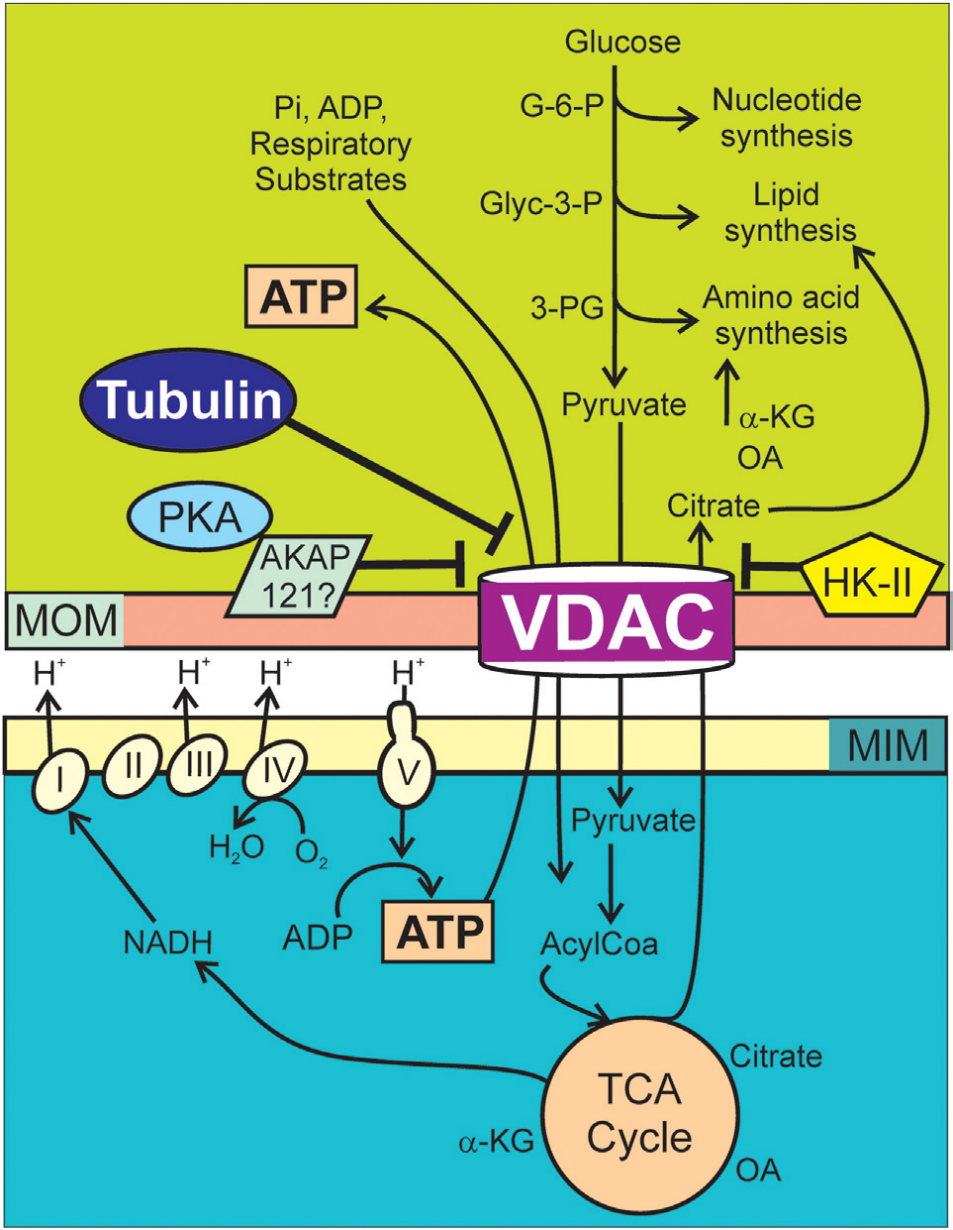
**Voltage-dependent anion channel (VDAC) regulation of Warburg metabolism**. Respiratory substrates, ADP, and Pi cross MOMs *via* VDAC and then MIMs *via* individual transporters. Respiratory substrates enter the Krebs cycle generating mostly NADH, which enters the respiratory chain (Complexes I–IV). Proton translocation from the matrix into the intermembrane space generates ΔΨ as oxygen is reduced to water. The F_1_F_0_ ATP synthase (Complex V) utilizes protons from the intermembrane space to drive the synthesis of ATP from ADP and Pi. Synthesis of nucleotides, lipids, and amino acids in the cytosol are supported by G-6-P, Glyc-3-P, and 3-PG originated in the catabolism of glucose and citrate, oxaloacetate, and α-ketoglutarate from the Krebs cycle. In cancer cells, high free tubulin blocks VDAC conductance. VDAC closure globally suppresses mitochondrial metabolism decreasing cytosolic ATP/ADP ratios. Low ATP/ADP ratios favor glycolysis. PKA phosphorylates VDAC increasing the sensitivity to tubulin inhibition and possibly stabilizes VDAC in a closed conformation by forming a complex with AKAP121. HK-II binds to VDAC and promotes VDAC closing. AKAP121, A-kinase anchor protein 121; α-KG, α-ketoglutarate; Glyc-3-P, glyceraldehyde 3-phosphate; G-6-P, glucose-6-phosphate; HK-II, hexokinase II; MIM, mitochondrial inner membrane; MOM, mitochondrial outer membrane; OA, oxaloacetate; PKA, protein kinase A; 3-PG, 3-phosphoglycerate.

The current consensus is that enhanced glycolysis in cancer cells is a source of carbon backbones for the synthesis of new macromolecules. Cell division requires a duplication of the biomass (lipids, proteins, and nucleic acids) before mitosis. Such an increase in biosynthesis could not be accomplished if glucose, glutamine, and fatty acids were fully oxidized in mitochondria. In the Warburg phenotype, incomplete breakdown of glucose to yield lactate provides precursors needed for biomass formation (22–26). Glucose-6-P, glyceraldehyde-3-P, and 3-phosphoglycerate derived from glucose are utilized in the synthesis of nucleotides, lipids, and amino acids, respectively. Elevated glycolytic flux also promotes generation of NADPH in the pentose phosphate pathway to be used in reductive biosynthesis and oxidation of NADH to NAD^+^ in the pyruvate to lactate step. In addition to glucose, cancer cells utilize glutamine and other fuels to generate biosynthetic precursors in the Krebs cycle including citrate used for lipid biosynthesis and oxaloacetate and α-ketoglutarate used for synthesis of some non-essential amino acids (Figure 1) (27). Thus, mitochondria not only generate energy but also play a biosynthetic role in the pro-proliferative Warburg phenotype. Recently, one-carbon metabolism, the set of reactions that transfer one carbon units from serine and glycine to donors, has been reported as critical components for *de novo* synthesis of purines and thymidylate during rapid tumor growth (28). Overall, the Warburg metabolism is a pro-proliferative phenotype that favors biosynthesis.

### Mitochondrial Metabolism, ATP/ADP Ratio, and Glycolysis

In differentiated cells, most of the respiratory substrates including pyruvate, fatty acyl-CoA, and amino acids are completely oxidized to CO_2_ and H_2_O by OXPHOS with a high yield of ATP. Newly synthesized ATP is transported to the cytosol through the adenine nucleotide translocator (ANT). A highly active mitochondria in a predominantly oxidative metabolism sustains cytosolic ATP/ADP ratios that can be 50–100 times higher compared to the mitochondrial matrix (29). High cytosolic ATP/ADP ratios suppress glycolysis through the inhibition of phosphofructokinase-1 (PFK-1) among other possible mechanisms. PFK-1, subjected to allosteric regulation, is strongly inhibited by ATP and activated by ADP and AMP (13, 30). By contrast, in cancer cells, a partial or complete suppression of mitochondrial metabolism determines a low ATP/ADP ratio that contributes to maintain enhanced glycolysis.

Proteins associated with the mitochondrial outer membrane (MOM) regulate both mitochondrial metabolism and glycolysis. Hexokinase II (HK-II), overexpressed in tumor cells and required for tumor initiation and tumor growth in mouse models, binds to VDAC1. HK-II stabilizes VDAC1 in a closed state, prevents apoptosis triggered by mitochondrial permeability transition (MPT), and favors glycolysis (31–35). Protein kinase A (PKA), known to form complexes in the MOM, phosphorylates voltage-dependent anion channel (VDAC) increasing the sensitivity to tubulin inhibition (36). PKA is also involved in the regulation of mitochondrial metabolism through the assembly of complexes with AKAP121, a protein of the family of A-kinase anchor proteins regulated by hypoxia and other cellular stresses (37, 38).

We recently proposed that inhibition of VDAC conductance by free tubulin and lack of activity of the ANT contribute to the suppression of mitochondrial metabolism and a low cytosolic ATP/ADP ratio in cancer cells (39–41). VDAC closing by free tubulin in cancer cells decreases the entrance of respiratory substrates to the mitochondrial matrix decreasing mitochondrial metabolism and lack of activity of ANT limits the ATP/ADP turnover (39, 41).

## VDAC Regulation of Mitochondrial Metabolism and Warburg Phenotype

### VDAC and Cellular Bioenergetics

The Warburg metabolism is sustained by chemical reactions occurring in interdependent cytosolic and mitochondrial compartments separated by the MOM (Figure 1). The MOM is not merely a physical separation but a functional barrier containing VDAC, a master key to globally modulate mitochondrial bioenergetics and the intracellular flow of energy (39, 40, 42). Crossing of polar metabolites through VDAC is determined mostly by the charge and size of the molecule (43, 44). By contrast, transport of polar metabolites between the matrix and the mitochondrial intermembrane space occurs through several specific transporters that catalyze the translocation of solutes across the mitochondrial inner membrane (MIM). Once inside the matrix, respiratory substrates enter the Krebs cycle generating mostly NADH that is further oxidized in the electron transport chain (ETC) to produce protons that are pumped to the intermembrane space at complexes I, III, and IV, creating a negative potential in the mitochondrial matrix and a proton motive force utilized by the ATP synthase (complex V) to generate ATP from ADP and Pi (Figure 1).

It has been proposed that VDAC closing could seal mitochondria and block mitochondrial metabolism becoming a “governor” of mitochondrial metabolism (31). Experimental evidence using single and double knockdown of VDAC1/2/3 showed that VDAC regulates mitochondrial metabolism in cancer cells as determined by mitochondrial ΔΨ, ATP production, and NADH generation (40). A consequence of dynamic mitochondrial “sealing–unsealing” is a lower or higher mitochondrial metabolism, a lower or higher cytosolic ATP/ADP ratio, and an enhancement or inhibition of glycolysis. Thus, VDAC regulation can function as a metabolic switch to promote or block OXPHOS and an adjustable rheostat with a range of operational levels that depend on the magnitude and duration of VDAC opening. An intriguing-related question is if genetic or pharmacological regulation of VDAC could be used not only to modulate oxidative metabolism but also to indirectly revert the Warburg phenotype.

### VDAC Structure and Regulation of Mitochondrial Metabolism

A protein with pore-forming activity first described in extracts of mitochondria from *Paramecium tetraurelia* (45) was initially called mitochondrial porin and later renamed VDAC (46). The voltage dependence of VDAC from different tissues and organisms (47) inserted in lipid bilayers was demonstrated by the closure induced by electrical potentials applied to membranes (48, 49). The relevance of unveiling the existence of a voltage-sensitive pore-forming protein in mitochondria was offset by the lack of clear evidence of a similar electrical potential across the MOM in intact cells. The role of VDAC voltage gating under physiological conditions is still controversial. It has been proposed that an estimated Donnan potential of −40 mV formed by impermeant charged species, mostly proteins, asymmetrically distributed across the membrane would be sufficient to promote VDAC closing in intact cells (50). Against this assumption, the presence of charged macromolecules at both sides of the MOM and a high cellular ionic strength makes not very likely the formation of a Donnan potential large enough to trigger VDAC gating in intact cells.

Voltage-dependent anion channel, present in all eukaryotic cells, is the most abundant protein in the MOM comprising three isoforms encoded by separate genes, VDAC1, VDAC2, and VDAC3. VDAC1 and VDAC2 are the main isoforms in most mammalian cells. The exception is VDAC3, especially abundant in testis (51, 52). VDAC in humans and mouse is a ~30 kDa protein enclosing an aqueous channel of ~3 nm internal diameter in the fully open state that allows the passage of molecules up to ~5 kDa (44, 53, 54). In the closed state, only small ions like Na^+^, K^+^, or Cl^−^ but not most anionic metabolites including respiratory substrates, ATP, ADP, and Pi permeate through VDAC. Structural studies using NMR and X-ray crystallography have shown VDAC1 as formed by 19 β-strands with the addition of an N-terminal sequence containing the only α-helical segments found in the protein (55, 56). A structural model proposes that the N-terminal residues of VDAC1 lying inside the pore parallel to the wall can move to the center of the channel blocking the passage of metabolites. Recently, the structure of VDAC2 in zebrafish has been solved showing a similar β barrel structure with 19 β-strands (57). Both VDAC1 and VDAC2 from eukaryotes have highly conserved biophysical properties including gating and selectivity (58). VDAC is the only known channel in the MOM that allows the passage of physiologically relevant respiratory substrates, ADP, and Pi into mitochondria. Thus, the probability of VDAC to remain in an open or close conformation is expected to have a substantial impact on mitochondrial metabolism and cellular bioenergetics.

For decades, research on mitochondrial bioenergetics has been mostly focused on the members of the mitochondrial carrier family SLC25 (solute carrier family 25) located in the MIM (59, 60). Proteins of the SLC25 family transport chemically diverse solutes including pyruvate, Pi, ADP, ATP, acylcarnitine, citrate, oxoglutarate, and glutamate across the MIM utilizing electrical, chemical, or electrochemical potential gradients. The activity of mitochondrial carriers is finely regulated to allow a sufficient flux of metabolites to adapt to different physiological demands (61). The availability of solutes to the carriers in the MIM depends on what metabolites are produced in the mitochondrial matrix mainly by the Krebs cycle and OXPHOS and what metabolites access the intermembrane space through VDAC in the MOM. Thus, regulation of VDAC opening is a unique element to control mitochondrial metabolism.

The initial consensus about VDAC being constitutively open as an “all-time open door” to the flux of metabolites between the mitochondrial matrix and the cytosol have been challenged by extensive research demonstrating modulation of VDAC conductance both *in vitro* and in intact cells. VDAC gating is regulated by several molecules including glutamate (62), NADH (63), hexokinase (64–66), and Bcl2 family members (67). VDAC phosphorylation by PKA, glycogen synthase 3β (GSK3β), and protein kinase C epsilon (PKCε) blocks or inhibits association of VDAC with other proteins, such as Bax and tBid (66, 68–73). As described above, PKA phosphorylates VDAC and decreases VDAC conductance by increasing the sensitivity to tubulin inhibition (36, 74), whereas GSK3β-mediated VDAC2 phosphorylation promotes channel opening (73). Some of these regulatory mechanisms were demonstrated *in vitro* but not in intact cells or tissues raising questions about the biological relevance of the findings.

Our group has reported two mechanisms of VDAC regulation in live cells, the closure of VDAC by free tubulin in cancer cells (9, 40) and after acute treatment of hepatocytes with ethanol (31, 75).

### VDAC and Free Tubulin in Cancer Cells

#### VDAC–Tubulin and Mitochondrial ΔΨ

**Figure 2:**
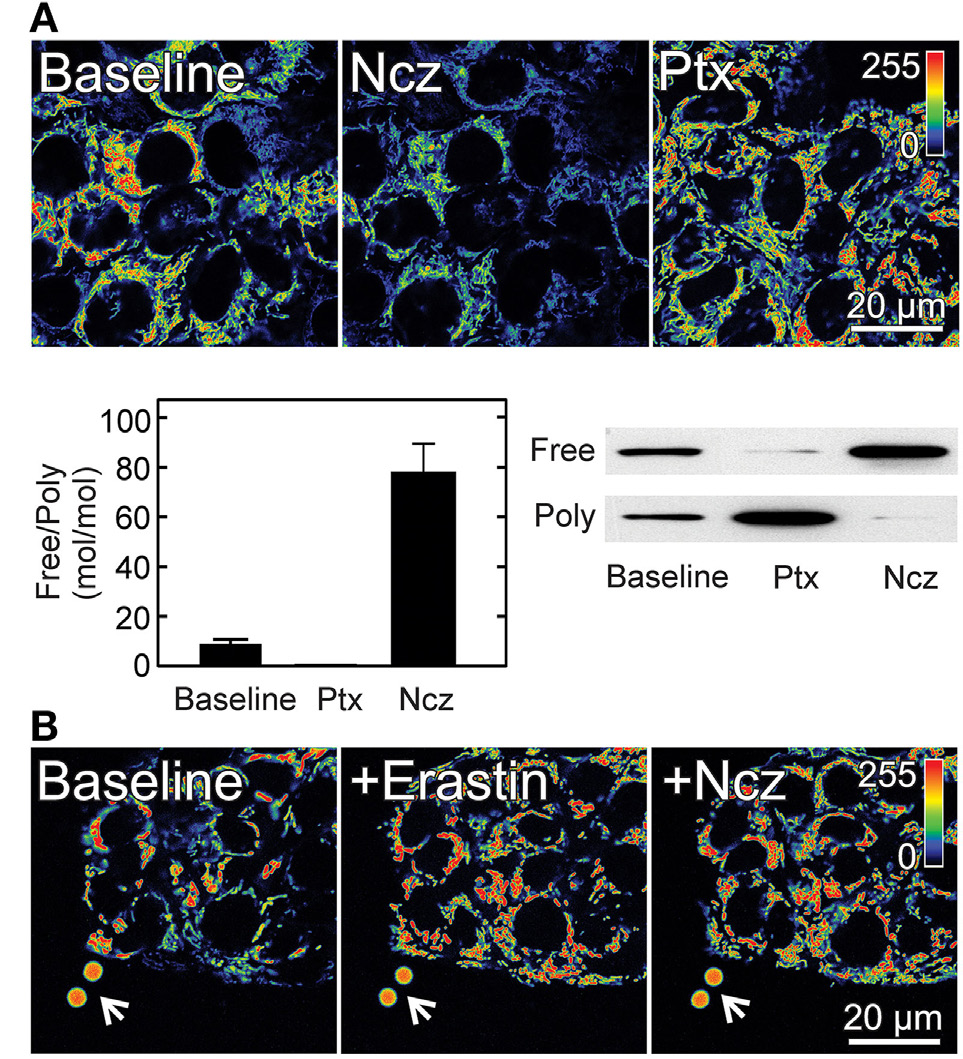
**Effect of free tubulin on mitochondrial membrane potential**. **(A)** HepG2 human hepatocarcinoma cells were loaded with the ΔΨ-indicating fluorophore tetramethylrhodamine methyl ester (TMRM). Nocodazole (Ncz; 10 µM), a microtubule destabilizer decreased ΔΨ, as shown by decreased fluorescence of TMRM (visualized in pseudocolor). Paclitaxel (Ptx; 10 µM), a microtubule stabilizer promoted mitochondrial hyperpolarization as evidenced by increased TMRM fluorescence. Free tubulin increased and decreased after Ncz and Ptx respectively as indicated by Western blotting of free and polymerized tubulin *p < 0.05. **(B)** Erastin (10 µM) increased ΔΨ in HepG2 cells. Mitochondria remained hyperpolarized after subsequent addition of Ncz (10 µM). Arrows identify 4 µm fiducial fluorescent beads. Adapted from Maldonado et al. (9, 40). Poly, polymerized.

In tumor cells, respiration and mitochondrial hydrolysis of glycolytic ATP sustain mitochondrial ΔΨ indicating a flux of metabolites including ATP between mitochondria and cytosol (9). We previously showed that maintenance of mitochondrial ΔΨ in cancer cells correlates inversely with the amount of cytosolic free tubulin. The microtubule destabilizers nocodazole and colchicine increased free tubulin and decreased mitochondrial ΔΨ. Conversely, the microtubule stabilizer paclitaxel promoted tubulin polymerization decreasing free tubulin and increasing mitochondrial ΔΨ [Figure 2; (9)]. These findings showed that free tubulin dynamically regulates mitochondrial metabolism as determined by measurements of mitochondrial ΔΨ. By contrast, in the non-proliferating rat hepatocyte, mitochondrial ΔΨ was relatively insensitive to changes in free tubulin levels possibly because tubulin polymerization is higher in hepatocytes compared to cancer cell lines. Our studies indicate that free tubulin is an endogenous regulator of mitochondrial ΔΨ in tumor cells but not in differentiated cells (9). The modulation of mitochondrial ΔΨ by tubulin led to the hypothesis that free tubulin closes VDAC and that VDAC closure contributes to the suppression of mitochondrial metabolism in the Warburg phenotype. This hypothesis was supported by previous work showing that heterodimeric αβ-tubulin closes VDAC inserted into lipid bilayers and also decreases respiration in isolated brain mitochondria, permeabilized synaptosomes, and cardiac myocytes (76, 77).

#### VDAC–Tubulin and the Cell Cycle

The free tubulin pool varies throughout the cell cycle especially decreasing during mitosis to allow the spindle formation. A potential implication of the changing levels of tubulin throughout the cell cycle is that the VDAC–tubulin-dependent suppression of mitochondrial metabolism be maximal when the cell is not dividing and free tubulin is relatively high and minimal during mitosis when free tubulin is low. VDAC–tubulin-dependent suppression of mitochondrial metabolism caused by high free tubulin would favor the pro-biosynthetic Warburg phenotype to support the generation of new macromolecules during the G1, S, and G2 phases of the cell cycle. In HeLa cells with a typical cell cycle of 20 h, the duration of G1, S, and G2 phases are 7, 9, and 2–3 h, respectively, whereas mitosis is completed in less than an hour. In unsynchronized HeLa, NIH3T3, and NCI-H292 cells, the G1 and S phase durations are the longest, whereas G2 and M accounts for only 15% of the cycle time (78). During mitosis, the free tubulin pool eventually polymerizes to form the spindle, releasing the inhibition on VDAC to increase mitochondrial metabolism and decrease glycolysis precisely when the energy demand is maximal. After mitosis, high free tubulin would close VDAC again and cells would return to a high glycolytic pro-proliferative phenotype until the next mitosis (42).

#### VDAC Isoforms and Tubulin

Voltage-dependent anion channel sensitivity to tubulin inhibition is isoform dependent. All cancer cells express the three VDAC isoforms in different proportions being VDAC1 and VDAC2 the major isoforms accounting for 90% of the total and VDAC3 the least abundant, usually around 10% (40, 79, 80). Knockdown of VDAC1, VDAC2, and VDAC3 in HepG2 cells decreased mitochondrial ΔΨ indicating that all VDAC isoforms contribute to ΔΨ formation. Noticeably, knockdown of VDAC3 caused the greatest drop in mitochondrial ΔΨ and also decreased the NAD(P)H/NAD(P)^+^ ratio, ATP, ADP, and total adenine nucleotides (40). Single and double knockdown of VDAC1, VDAC2, and VDAC3 in the different possible combinations blunted the suppression of mitochondrial ΔΨ induced by free tubulin and suggested that VDAC1 and VDAC2 are the isoforms closed by tubulin in wild-type cells (40). Electrophysiology studies demonstrated voltage gating and response to dimeric αβ-tubulin almost identical in VDAC isolated from wild-type HepG2 cells compared to VDAC isolated from liver and heart mitochondria. VDAC1 and VDAC2 isolated from double knockdown VDAC2/3 and VDAC1/3 HepG2 cells, respectively, inserted in lipid bilayers were almost equally sensitive to tubulin inhibition. By contrast, VDAC3 was insensitive even at tubulin concentrations fivefold higher than those used to inhibit VDAC1 and VDAC2 (40). The knockdown studies supported the conclusion that VDAC3, at least in HepG2 cells, is constitutively open and VDAC1 and VDAC2 are totally or partially blocked by free tubulin.

## Tumor Metabolic Flexibility: Bioenergetics in Dynamic Equilibrium

The metabolic control analysis proposes to analyze individual chemical reactions and even activities of rate-limiting step enzymes in the context of interconnected and interdependent groups of reactions to evaluate the impact of a change in one component of the system on the global function (81, 82). The top-down metabolic control analysis or modular analysis group chemical reactions in blocks to identify regulators of metabolism based on the supply or consumption of a certain intermediate (83). This approach showed that respiration in intact rat hepatocytes was mostly controlled by ATP synthesis, and the rest of the control was distributed between the proton leak and the reactions that sustain ΔΨ (84). Metabolic control analysis may eventually be a tool to better understand the bioenergetics and metabolic consequences of the switching between glycolysis and OXPHOS in tumor cells.

The predominance of a glycolytic or oxidative metabolism in cancer cells is not determined only by the genetic program but subjected to temporary and long-term epigenetic changes. Changes in the relative contribution of glycolysis and OXPHOS to the cellular ATP generation in tumor cells is triggered by different stimuli including the level of oxygenation, amount and type of nutrients available, proximity to neoformed or mature blood vessels, release of soluble factors including lactate from neighboring cancerous or non-cancerous cells, and the stage of the cell cycle.

In MCF-7 and HeLa cells, prolonged hypoxia increased glycolysis but only in MCF-7 the OXPHOS flux decreased even though both cell lines predominantly depended on OXPHOS for ATP supply (85). The variability in the response to hypoxia may depend on cell type, time of exposure to low levels of oxygen, and environmental conditions. In solid tumors with a heterogeneous perfusion, OXPHOS can still produce ATP considering that hypoxic tumor cells are exposed to <2% of oxygen and the ETC can function optimally at oxygen levels as low as 0.5%. Under those conditions, even if pyruvate utilization is compromised, mitochondria from tumor cells can utilize glutamine as an energy source so actually both glycolysis and OXPHOS can sustain tumor growth (86). Inadequate blood flow as it occurs during imperfect angiogenesis not only causes hypoxia but also insufficient glucose supply. Long-term culture in glucose-deprived medium led to increased OXPHOS and decreased glycolysis in two breast cancer cell lines confirming the influence of the microenvironment on the bioenergetics profile (87). The switch from aerobic glycolysis to OXPHOS was also observed in mantle cell lymphoma cells cultured in glucose-free media (88). If glucose or glutamine are limited, still tumor cells can utilize a wide variety of substrates to support the energetic needs (89) including asparagine (90), leucine (91), arginine (92), methionine (93), valine (94), cysteine (95), lactate (96, 97), acetate (98, 99), and even vesicle-driven pathways to uptake proteins and lipids from the environment (100). Inhibition of complex I by piericidin A or complex III by antimycin in myoblasts led to a compensatory increase in uptake and glucose consumption. In these cells, cellular ATP production with or without OXPHOS inhibition was similar indicating that suppression of OXPHOS was quickly and fully compensated by the increase in glycolytic ATP generation (101).

## VDAC–Tubulin Antagonism, Oxidative Stress, and Reversal of Warburg Phenotype

### The VDAC–Tubulin Interaction: A Pharmacological Target

In the last decades, attempts to inhibite glycolysis to decrease tumor growth have been a major focus of research on tumor metabolism (102, 103). Only more recently, mitochondrial metabolism emerged as another option for the development of new cancer treatments (104, 105). The lower prevalence of certain types of cancer in patients taking the antidiabetic drug metformin raised the interest on mitochondria as a potential target to suppress tumor growth (106, 107). Although the mechanism of action of metformin is not entirely clear, it has been shown to decrease OXPHOS by inhibiting complex I of ETC, to activate AMPK, to inhibit the mammalian target of rapamycin, and to interfere on folate metabolism (108). Other approaches to inhibit mitochondrial metabolism have included the use of glutaminase inhibitors (109), etomoxir to inhibit the carnitine *O*-palmitoyltransferase 1 and prevent subsequent mitochondrial fatty acid oxidation (110), the compound VLX600 to inhibit OXPHOS and reduce colon cancer tumor growth (111) and the antibiotic tigecycline to inhibit mitochondrial protein translation and decrease tumor growth in several experimental models of leukemia (112). Whereas most of treatments aim to decrease mitochondrial metabolism, the pyruvate analog dichloracetate, that causes cell death in several cancer cell lines and in some *in vivo* models, increases mitochondrial metabolism by activating pyruvate dehydrogenase and the subsequent delivery of pyruvate to mitochondria (113).

Our initial findings, showing that VDAC regulates mitochondrial metabolism and free tubulin closes VDAC, suggested that antagonizing the VDAC–tubulin interaction could be a novel pharmacological approach to increase OXPHOS and to revert Warburg metabolism. We showed that the small molecule erastin antagonizes the inhibitory effect of free tubulin on VDAC (40). Erastin, found in a synthetic lethal chemical screening in human cells engineered to harbor small T oncoprotein and the oncogenic allele of HRAS, the v-Ha-ras Harvey rat sarcoma viral oncogene homolog RAS^v12^, selectively induced non-apoptotic cell death (114). A lung carcinoma cell line harboring the v-Ki-ras2 Kirstej rat sarcoma viral oncogene homolog and other cell line containing an activating V600E mutation in v-raf-murine sarcoma viral oncogene homolog B1 (BRAF) were moderately sensitive to erastin. Erastin-induced cell death was not prevented by pan-caspase inhibitors, but it was blocked by antioxidants including α-tocopherol and butylated hydroxytoluene (114). It has been proposed that erastin binds to VDAC2 and VDAC3 leading to mitochondrial dysfunction, release of oxidative species, and cell death in cells with activated RAS-RAF-MEK signaling (115).

In wild-type HepG2 cells, erastin hyperpolarizes mitochondria and completely abrogates and reverses mitochondrial depolarization induced by microtubule destabilizers indicating that erastin both prevents and reverses free tubulin-dependent inhibition of ΔΨ formation [Figure 2; (40)]. Further studies of VDAC from wild-type HepG2 inserted into planar lipid bilayers showed that erastin added after tubulin completely blocked the decrease in VDAC conductance induced by tubulin. Erastin restored the voltage dependence to a response almost identical to that observed in the absence of tubulin. In addition, erastin added alone in the absence of tubulin did not modify the current–voltage profile of VDAC indicating that the effect of erastin was specific for tubulin-dependent inhibition of conductance (40). A new group of lead compounds identified in a high throughput cell-based screening, similar to erastin hyperpolarized mitochondria in the presence of high levels of free tubulin after treatment with nocodazole.

### VDAC Opening, Formation of Reactive Oxygen Species (ROS), and Mitochondrial Dysfunction

Voltage-dependent anion channel controls the flux of respiratory substrates entering the Krebs cycle. Electron pairs from NADH flow down the ETC to the final acceptor O_2_. Single electrons also leak from complexes I, II, and III to form the superoxide anion $\left({\text{O}}_{2}{•}^{-}\right)$ (116). Complex I (site I_Q_), complex II (site II_F_), and complex III (site III_Qo_) have the highest capacity of ROS production among the seven major mitochondrial sites that produce ROS in mammals (117–119). Although there are other mitochondrial and non-mitochondrial sources of ROS formation, the mitochondrial ETC is quantitatively the most important (120).

Voltage-dependent anion channel opening leads to increased activity of the ETC chain and increased generation of the free radical ${\text{O}}_{2}{•}^{-}$ that is rapidly converted to H_2_O_2_ by superoxide dismutases located in the mitochondrial matrix (manganese-containing enzyme MnSOD or SOD2) and the cytosol (copper-and-zinc-containing enzyme CuZnSOD or SOD1) (121). Subsequently, H_2_O_2_ accepting one electron from free Fe^2+^ by the Fenton reaction produces the highly reactive hydroxyl radical (OH•^−^). ${\text{O}}_{2}{•}^{-}$ formed at complexes I and II diffuse to the matrix, whereas ${\text{O}}_{2}{•}^{-}$ generated at complex III diffuse both to the matrix and to the intermembrane space from where it is released to the cytosol through VDAC (122–124). Both ${\text{O}}_{2}{•}^{-}$ and especially the highly reactive OH•^−^ are damaging for cells. By contrast, H_2_O_2_, a non-radical molecule and the least reactive of ROS, diffuse across membranes and act as a secondary messenger modulating pro-proliferative and pro-survival pathways without disrupting redox homeostasis (125, 126).

Cancer cells have higher basal levels of ROS compared to differentiated cells as evidenced in cell lines by increased H_2_O_2_ formation and in animal models and human tissues by increased oxidative-dependent DNA modifications and 4-hydroxy-2-non-enal modified proteins (127–130). However, higher levels of ROS are balanced off by the higher content of scavenging enzymes and antioxidants including SODs, catalase that catalyzes the conversion of H_2_O_2_ to H_2_O and O_2_, and the glutathione system that reduces disulfide bonds of cytoplasmic protein to cysteines (131–134). It has been proposed, although not established experimentally, that different ROS levels can be cytostatic, promote tumorigenesis, or be cytotoxic (132, 133, 135). Oxidative stress has been reported to induce mitochondrial dysfunction, cancer cell cycle arrest, senescence, apoptosis, or necrosis (131).

H_2_O_2_ and to a less extent ${\text{O}}_{2}{•}^{-}$ react with intramitochondrial components but are also released from mitochondria to affect cytosolic proteins and other organelles. By contrast, the reactions with the highly reactive OH•^−^ are dependent on the rate of diffusion and almost completely restricted to mitochondria. OH•^−^ and ${\text{O}}_{2}{•}^{-}$ inactivate mitochondrial proteins including NADH dehydrogenase, NADH oxidase, and ATP synthase (136). When the antioxidant capacity is exceeded, ROS accumulation in the mitochondrial matrix also damages lipids and transporters in the MIM and mitochondrial DNA. Peroxidation of the polyunsaturated fatty acyl chains of cardiolipin, a phospholipid found exclusively in the MIM, is an early event in the intrinsic apoptotic pathway (137). Cytosolic ROS activate signaling pathways that cause mitochondrial dysfunction including the members of the MAPK family of serine/threonine kinases especially the c-Jun N-terminal kinases (JNK), the extracellular signal-regulated kinase (ERK 1/2), and p38 (138, 139). JNK activation caused by oxidative stress promotes mitochondrial dysfunction by a poorly understood mechanism although the onset of MPT has been proposed to be triggered by activated JNK translocated to mitochondria (140, 141).

Because ROS are by-products of multiple chemical reactions not generated by specific pathways, ROS concentration depends on the regulation of ROS-forming reactions. Drug-induced VDAC opening in tumor cells is expected to increase mitochondrial ROS formation and promote oxidative stress. The accumulation of ROS above a threshold should eventually break the dynamic equilibrium between ROS generation and antioxidant systems leading to cytotoxic effects.

### The Metabolic Double Hit: Oxidative Stress and Anti-Warburg Effect

Tumor heterogeneity, increasingly recognized as an important feature in cancer biology, is a complicating factor for successful chemotherapy because genetic and metabolic differences in cancer cells even inside the same tumor affect the response to cancer treatments (142–145). Although tumor cells have different metabolic signatures, most of them display some level of enhanced glycolysis indicating a differential contribution of VDAC closure to the suppression of mitochondrial metabolism. Increased OXPHOS and subsequent ROS generation by drug-induced VDAC opening should affect most of the cancer cells considering that enhanced glycolysis and suppression of mitochondrial metabolism is a characteristic of tumors (11–14).

**Figure 3:**
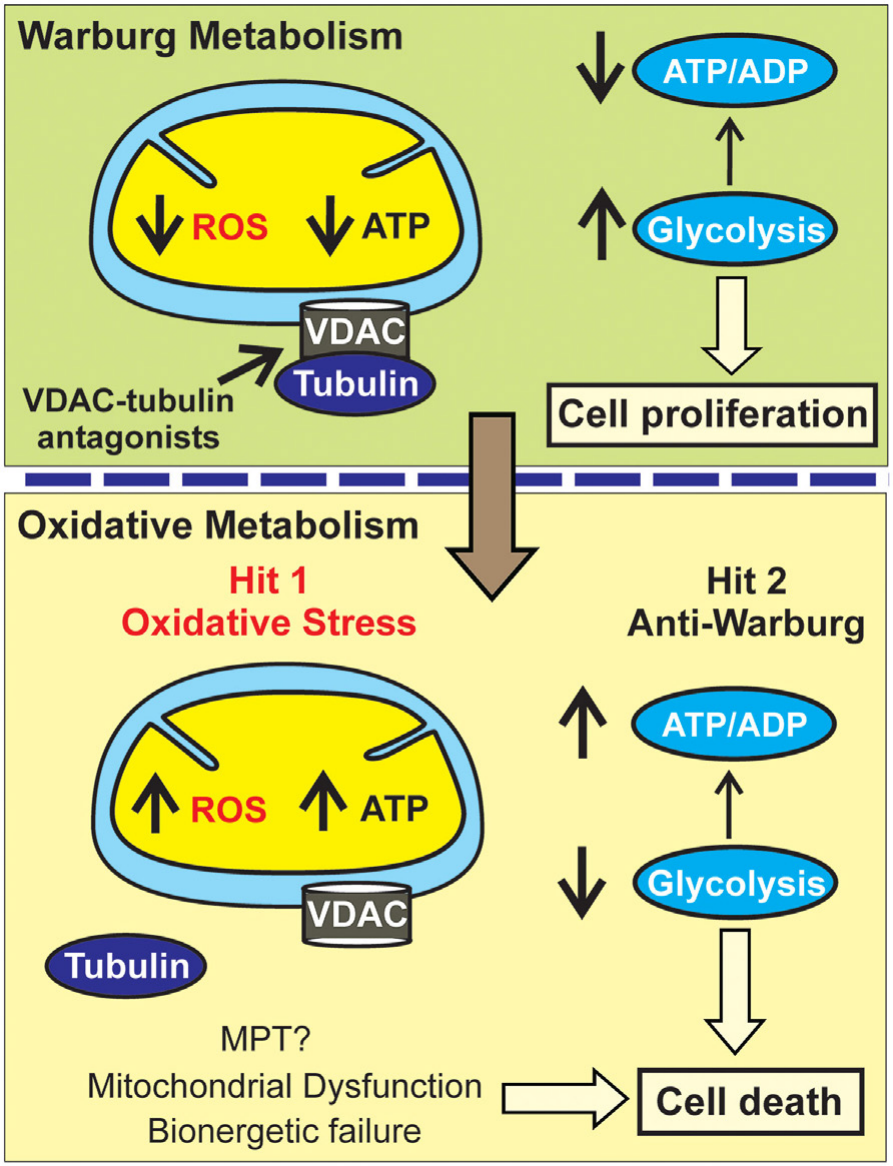
**Metabolic double hit after VDAC opening**. In the Warburg metabolism, free tubulin closes VDAC resulting in low mitochondrial reactive oxygen species (ROS) generation and ATP formation leading to a low ATP/ADP ratio and enhanced glycolysis. VDAC-tubulin antagonists by opening VDAC promote a switch to an oxidative metabolism characterized by increased ROS formation (Hit 1: oxidative stress). Increased mitochondrial metabolism after VDAC-tubulin antagonists also increases ATP formation and promotes a high ATP/ADP ratio that inhibits glycolysis (Hit 2: anti-Warburg effect). MPT, mitochondrial permeability transition.

Blockage of the inhibitory effect of tubulin on VDAC is expected to trigger two separate but concurrent effects: the increase in ROS formation leading to oxidative stress (first hit) and the reverse of the Warburg metabolism caused by the increase in OXPHOS and ATP synthesis with the subsequent decrease in glycolysis (second hit) (Figure 3). Both effects will likely be quantitatively more important in highly glycolytic tumors. A potential implication for high vs low glycolytic cells is that oxidative stress may promote more cell killing in highly glycolytic tumors with a lower mitochondrial metabolism and relatively low basal ROS production. By contrast, the reversal of the Warburg effect could be more relevant to those highly glycolytic cells that survive the initial hit caused by oxidative stress and continue proliferating or to the low glycolytic cells with a presumably higher basal level of ROS in which the anti-Warburg effect and not a further increase in ROS would be key to stop cell proliferation.

We propose that oxidative stress after VDAC–tubulin antagonists activates stress kinases especially JNK eventually leading to mitochondrial dysfunction, possibly the onset of MPT, and bioenergetic failure. MPT is a non-selective permeabilization of the MIM that causes a loss of ΔΨ and ATP synthesis, mitochondrial swelling, rupture of the MOM, and cytochrome *c* release resulting in cell death (146, 147). MPT has been proposed to be mediated by the irreversible opening of the permeability transition pore complex (PTPC), a multiprotein pore assembled with core components of both the MOM and the MIM. VDAC, ANT, cyclophilin D, and the subunit c of the F_1_F_0_ ATP synthase among other mitochondrial proteins have been included as PTPC-forming proteins. Despite of research efforts devoted to unequivocally identify the components of the PTPC, the molecular identity of the pore remains a matter of debate (148). VDAC, initially considered a main component of the pore, has been shown to be dispensable for the onset of MPT. Oxidative stress, a well-known inducer of MPT (146, 149, 150), promotes MPT even in cells knockout for all VDAC isoforms (151).

In our current model of cell death after VDAC–tubulin antagonists, oxidative stress causes mitochondrial dysfunction and bioenergetic failure and increased OXPHOS by increasing ATP causes a compensatory decrease of glycolysis independent of any potential role of VDAC in PTPC complex formation. A therapeutic advantage of VDAC–tubulin antagonists would be the selectivity to kill only cancer cells because in non-proliferating cells VDAC is constitutively open and not regulated by free tubulin (9, 42). In summary, the cytotoxic effects of VDAC–tubulin antagonists would follow a “two-hit” model of metabolic intervention characterized by a promotion of oxidative stress and an anti-Warburg effect (Figure 3).

## Concluding Remarks

The VDAC–tubulin interaction in cancer cells operates as a metabolic switch susceptible of pharmacological inhibition. Antagonism of the inhibitory effect of free tubulin on VDAC opens a new avenue in metabolism-oriented chemotherapy. Unlike other cancer treatments that inhibit specific pathways with restricted effects, VDAC opening exerts a global influence on mitochondrial metabolism which indirectly modulates glycolysis. Disruption of the switch causes a “two-hit” effect, the oxidative stress leading to mitochondrial dysfunction and a compensatory anti-Warburg decrease in glycolysis that turn cells into a non-proliferative phenotype. VDAC-dependent oxidative stress is expected to promote cell killing in highly glycolytic cells and to cause non-lethal cell damage in the less glycolytic tumor types. Reversal of the Warburg effect complements the effects of oxidative stress and decreases or stops cell proliferation in cells that survive oxidative stress or in those with relatively low glycolysis. In summary, we unveil a new pharmacological target with the capability of exploiting the metabolic flexibility of tumors to turn a pro-proliferative phenotype into a cytotoxic and non-proliferative mitochondrial-dependent metabolism.

## Author Contributions

The author prepared the whole manuscript and figures.

## Conflict of Interest Statement

The author declares that the research was conducted in the absence of any commercial or financial relationships that could be construed as a potential conflict of interest.
